# Supplementary material for: Comparative multi-tissue profiling reveals extensive tissue-specificity in transcriptome reprogramming during thermal adaptation
Source: eLife. 2022 May 17;11:e78556. doi: 10.7554/eLife.78556 (PMC9113744; doi:10.7554/eLife.78556)
Supplement: Transparent reporting form [file elife-78556-transrepform1.docx]

***eLife’s* transparent reporting form**

We encourage authors to provide detailed information *within their submission* to facilitate the interpretation and replication of experiments. Authors can upload supporting documentation to indicate the use of appropriate reporting guidelines for health-related research (see [EQUATOR Network](http://www.equator-network.org/%20)), life science research (see the [BioSharing Information Resource](https://biosharing.org/)), or the [ARRIVE guidelines](http://www.plosbiology.org/article/info:doi/10.1371/journal.pbio.1000412) for reporting work involving animal research. Where applicable, authors should refer to any relevant reporting standards documents in this form.

If you have any questions, please consult our Journal Policies and/or contact us: [editorial@elifesciences.org](mailto:editorial@elifesciences.org).

**Sample-size estimation**

- You should state whether an appropriate sample size was computed when the study was being designed
- You should state the statistical method of sample size computation and any required assumptions
- If no explicit power analysis was used, you should describe how you decided what sample (replicate) size (number) to use

Please outline where this information can be found within the submission (e.g., sections or figure legends), or explain why this information doesn’t apply to your submission:

Sample sizes were chosen base on previous mice experiments with power calculations of 0.8, designed to detect physiologically relevant differences.

**Replicates**

- You should report how often each experiment was performed
- You should include a definition of biological versus technical replication
- The data obtained should be provided and sufficient information should be provided to indicate the number of independent biological and/or technical replicates
- If you encountered any outliers, you should describe how these were handled
- Criteria for exclusion/inclusion of data should be clearly stated
- High-throughput sequence data should be uploaded before submission, with a private link for reviewers provided (these are available from both GEO and ArrayExpress)

Please outline where this information can be found within the submission (e.g., sections or figure legends), or explain why this information doesn’t apply to your submission:

1. RNA sequencing was performed once which is common for transcriptomics studies.

2. Each sample represents biological replicate, where RNA from different organs of mice are taken as specified in the manuscript (eg. Figure 3A).

3. Information on replicates is shown in the plots of Figure 3C & 3E.

4. In the whole study, one outlier was removed from brain samples after thorough statistical testing. Specifically, in the PCA plot, one Brain RT sample clustered differently from all other RT and CE samples within the principle component 1, which explained 98% variability of the data. Therefore, this sample was removed from the study. The sequencing data for that sample is provided in the publicly available reposted data. No other outliers were detected.

6. The high-throughput sequence data are now provided and available in the requested format, see data availability, page 31.

**Statistical reporting**

- Statistical analysis methods should be described and justified
- Raw data should be presented in figures whenever informative to do so (typically when N per group is less than 10)
- For each experiment, you should identify the statistical tests used, exact values of N, definitions of center, methods of multiple test correction, and dispersion and precision measures (e.g., mean, median, SD, SEM, confidence intervals; and, for the major substantive results, a measure of effect size (e.g., Pearson's r, Cohen's d)
- Report exact p-values wherever possible alongside the summary statistics and 95% confidence intervals. These should be reported for all key questions and not only when the p-value is less than 0.05.

Please outline where this information can be found within the submission (e.g., sections or figure legends), or explain why this information doesn’t apply to your submission:

1.The statistical methods are explained throughout the text, legends and methods.

2. Raw data for all the analysis and figures are provided as supplementary excel tables and are provided in an online application at https://metlabomics.unige.ch/Search

3. For each experiment the specified information are provided.

4. All the *P*-values are reported in the supplementary excel tables, or in the text when appropriate.

(For large datasets, or papers with a very large number of statistical tests, you may upload a single table file with tests, Ns, etc., with reference to sections in the manuscript.)

**Group allocation**

- Indicate how samples were allocated into experimental groups (in the case of clinical studies, please specify allocation to treatment method); if randomization was used, please also state if restricted randomization was applied
- Indicate if masking was used during group allocation, data collection and/or data analysis

Please outline where this information can be found within the submission (e.g., sections or figure legends), or explain why this information doesn’t apply to your submission:

Mice were randomly distributed into groups upon arrival to our animal facility.

**Additional data files (“source data”)**

- We encourage you to upload relevant additional data files, such as numerical data that are represented as a graph in a figure, or as a summary table
- Where provided, these should be in the most useful format, and they can be uploaded as “Source data” files linked to a main figure or table
- Include model definition files including the full list of parameters used
- Include code used for data analysis (e.g., R, MatLab)
- Avoid stating that data files are “available upon request”

Please indicate the figures or tables for which source data files have been provided:

All additional data files in form of tables are provided in the manuscript.

The used data files for all figures are also provided as Supplementary excel tables. All figures can be reproduced using the data that is already provided.

The R pipeline to reproduce all the results is provided via GitHub, specified in the methods section of the manuscript.

The raw counts from the RNA-seq data, and the code for the bioinformatics pipeline developed for this study have been made freely available at (github.com/Nhadadi/Mouse_AllTissue_Transcriptomics). The accession number for RNA-seq data reported in this paper is GSE198046.
